# Pan-cancer illumination of TRIM gene family reveals immunology regulation and potential therapeutic implications

**DOI:** 10.1186/s40246-022-00441-9

**Published:** 2022-12-02

**Authors:** Yueying Gao, Tao Pan, Gang Xu, Si Li, Jing Guo, Ya Zhang, Qi Xu, Jiwei Pan, Yanlin Ma, Juan Xu, Yongsheng Li

**Affiliations:** 1 Hainan Provincial Key Laboratory for Human Reproductive Medicine and Genetic Research, Reproductive Medical Center, National Center for International Research, The First Affiliated Hospital of Hainan Medical University, Hainan Medical University, Haikou, 571199 Hainan China; 2 College of Biomedical Information and Engineering, Hainan Medical University, Haikou, 571199 Hainan China; 3 College of Bioinformatics Science and Technology, Harbin Medical University, Harbin, 150081 Heilongjiang China

**Keywords:** TRIM genes, Pan-cancer, Immune pathways, Immunotherapy response

## Abstract

**Background:**

The tripartite motif (TRIM) proteins function as important regulators in innate immunity, tumorigenesis, cell differentiation and ontogenetic development. However, we still lack knowledge about the genetic and transcriptome alterations landscape of TRIM proteins across cancer types.

**Methods:**

We comprehensively reviewed and characterized the perturbations of TRIM genes across > 10,000 samples across 33 cancer types. Genetic mutations and transcriptome of TRIM genes were analyzed by diverse computational methods. A TRIMs score index was calculated based on the expression of TRIM genes. The correlation between TRIMs scores and clinical associations, immune cell infiltrations and immunotherapy response were analyzed by correlation coefficients and gene set enrichment analysis.

**Results:**

Alterations in TRIM genes and protein levels frequently emerge in a wide range of tumors and affect expression of TRIM genes. In particular, mutations located in domains are likely to be deleterious mutations. Perturbations of TRIM genes are correlated with expressions of immune checkpoints and immune cell infiltrations, which further regulated the cancer- and immune-related pathways. Moreover, we proposed a TRIMs score index, which can accurately predict the clinical outcome of cancer patients. TRIMs scores of patients are correlated with clinical survival and immune therapy response across cancer types. Identifying the TRIM genes with genetic and transcriptome alterations will directly contribute to cancer therapy in the context of predictive, preventive, and personalized medicine.

**Conclusions:**

Our study provided a comprehensive analysis and resource for guiding both mechanistic and therapeutic analyses of the roles of TRIM genes in cancer.

**Supplementary Information:**

The online version contains supplementary material available at 10.1186/s40246-022-00441-9.

## Background

Tripartite motif (TRIM) family proteins, most of which have E3 ubiquitin ligase activities, have various functions in cellular processes including apoptosis, innate immunity, autophagy, and carcinogenesis [1]. The structure of TRIM proteins is highly conserved, and *N*-terminal mainly includes RING-finger domain, zinc-finger domain named B box (B1/B2 box) and coiled coil region [1, 2]. Perturbations of ubiquitylation events induced by TRIM proteins have been involved in cancer progression and metastasis.

TRIM proteins have been demonstrated to play an important role in various cancer-related processes. They are participated in biological processes such as viral immunity, inflammatory response, autophagy and tumor growth, and regulate cytokines related to immune diseases [3, 4]. TRIM proteins may regulate many oncogenes and tumor suppressors by changing the stability of ubiquitin ligase, thus affecting the progression of cancer [5]. For example, TRIM proteins can interact with P53 and promote its ubiquitination and degradation, and then affect the activity of P53 related genes and pathways [6–8]. Moreover, mutations or deletions of TRIM proteins may also be associated with a variety of cancers. For instance, the lack of TRIM72 could increase the invasive of cancers and lead to the increased level of some oncogenes [9]. Mutations in TRIM26 also resulted in decreased immune response, which in turn increased the risk of proliferation and metastasis of cancer [10]. Although these results suggest the genetic alterations of TRIM proteins across cancer types, the genetic and transcriptome landscapes of TRIM proteins are unknown in cancer.

The innate immune system is the first line of host defense against microbial infection, and protein post-translational modifications (PTMs) are important mechanisms to activate immune signaling pathways. Therefore, TRIM protein with ubiquitin ligase activity may play an important role in regulating innate immunity [11, 12]. Previous studies have indicated that TRIM proteins may regulate nuclear factor κB (NF-κB) and IFN regulatory factor (IRF) families of transcription factors via regulating innate immune signals in defense against pathogens and immune-related diseases [4, 13]. TRIM60, TRIM41 and TRIM38 have been demonstrated to regulate innate immune and inflammatory pathways [14, 15]. Furthermore, several TRIM proteins are abnormally expressed in many cancers and may be involved in the regulatory process of cancer. The up-regulation of TRIM59 promotes the ubiquitination and degradation of P53, inhibits the expression of downstream molecules, and leads to the inactivation of p53 signaling pathway, which may promote the proliferation and metastasis of gastric cancer [6]. In the mouse xenogeneic model with TRIM59 knockout, down-regulation of cyclin was found. TRIM59 may promote the proliferation and metastasis of non-small cell lung cancer, hepatocellular carcinoma and prostate cancer by regulating cyclin related proteins [16–18]. It was also found that down-regulation of TRIM59 enhanced the chemosensitivity of esophageal cancer to cisplatin [19]. However, we still lack a comprehensive knowledge about the regulation of TRIM proteins on tumor immune microenvironments.

To addresses these gaps of TRIM proteins in cancer, we comprehensively reviewed and characterized the perturbations of TRIM genes across > 10,000 samples across 33 cancer types in this study. We found that somatic mutations in TRIM genes and protein levels frequently emerge in a wide range of tumors and greatly affect the expression of TRIM genes in cancer. In particular, we found that somatic mutations located in TRIM protein domains are likely to be deleterious mutations. Perturbations of TRIM genes are correlated with expressions of immune checkpoints and immune cell infiltrations. The genes correlated with TRIM genes are significantly enriched in the cancer- and immune-related pathways. Moreover, we proposed a TRIMs score index based on the expression of TRIM genes. We found that TRIMs scores can accurately predict the clinical outcome of cancer patients. TRIMs scores of patients are correlated with clinical survival and immune therapy response across cancer types. All the results provided a comprehensive review and resource for guiding both mechanistic and therapeutic analyses of the roles of TRIM genes in cancer.

## Methods

### Collection of TRIM genes

We collected the TRIM family proteins, most of which contain RING-finger domains, from one recent study [1]. In total, 77 members in TRIM family were obtained in this study and genes were further classified into sub-families based on gene annotations. In addition, we obtained the domain coordinates in TRIMs proteins from UniProt [20] (https://www.uniprot.org/) and Pfam (http://pfam.xfam.org/) [21].

### Somatic mutations across cancer types

Genome-wide somatic mutation datasets across 33 cancers were obtained from The Cancer Genome Atlas (TCGA, https://portal.gdc.cancer.gov/). We explored the mutation frequency of TRIM proteins in 33 cancer types. Based on the domains of TRIM proteins, all members of TRIM family were classified into 12 subtypes.

### Mutational effects of TRIMs

We first calculated the mutation frequency of each TRIM subtype *i* in cancer *j* as follows:

$${\text{Mut}}_{{{\text{SubFamily}}\left( {i,j} \right)}} = \frac{{m\left( {i,j} \right)}}{M\left( j \right)} \times \frac{{n\left( {i,j} \right)}}{N\left( i \right)}$$

where $m\left( {i,j} \right)$ is the number of mutated samples of TRIM subtype *i* in cancer *j*. $M\left( j \right)$ is the number of all mutated samples in cancer *j*. $n\left( {i,j} \right)$ is the number of members of TRIM subtype *i* mutated in cancer *j*. $N\left( i \right)$ is the number of all members of TRIM subtype *i*. The mutation frequency not only takes into account the number of mutated samples in each cancer, but also the number of members in TRIM subtypes.

Based on the mutations and domains of TRIM proteins, we next calculated the frequency of mutations occurring in domains as follows:

$$f\left( {a,j} \right) = \frac{{d\left( {a,j} \right)}}{N\left( j \right)}$$

where $d_{{\left( {a,j} \right)}}$ refers to the number of mutated samples in domain *a* in cancer *j*, $N\left( j \right)$ is the number of mutated samples in cancer *j*.

In addition, we investigated the functional impacts of somatic mutations by ANNOVAR [22]. SIFT [23], PolyPhen-2 [24], CADD [25] and conservation scores were used to evaluate the mutational impacts on the structure and function of proteins. Wilcoxon rank-sum test was used to evaluate the differences between the mutations located in domain and other regions of TRIM genes.

### Prioritization of domains enriched somatic mutations

We applied ROI-Driver for identification of TRIM genes which enriched somatic mutations in protein domain regions [26]. For each domain in TRIM proteins, we assumed that the observed number of mutations for a domain follows a binomial distribution [27]. The binomial is $\left( {N,p_{ri} } \right)$, in which *N* is the total number of mutations observed in one gene and $p_{ri}$ is the expected mutation rate for the domain. We next calculated the *p* value, which is the probability of observing ≥ *k* mutations in the domain out of *N* total mutations observed in this gene:

$$P\left( {X \ge k} \right) = 1 - P(X < k) = 1 - \mathop \sum \limits_{x = 0}^{k - 1} \left( \frac{N}{x} \right)p_{ri}^{x} \left( {1 - p_{ri} } \right)^{N - x}$$

where $p_{ri} = \frac{{L_{{{\text{domain}}}} }}{{L_{{\text{g}}} }}$, and $L_{{{\text{domain}}}}$ represents the length of the domain and $L_{{\text{g}}}$ is the length of gene. In addition, we calculated the enrichment ratio for each domain as follows:

$$E_{{{\text{domain}}}} = \frac{k}{{N*L_{{{\text{domain}}}} /L_{{\text{g}}} }}$$

The *p* values were adjusted, and domains with *p* adjusted < 0.05 and *E* > 2 were identified as significant domains. Only domains with three or more mutations were analyzed.

### Gene expression profiles across cancer types

The gene expression datasets of cancer patients were also downloaded from TCGA (https://portal.gdc.cancer.gov/). The expressions between different samples from the same patient are taken as the average. Gene expression was measured by Fragments Per Kilobase of exon model per Million mapped fragments (FPKM). Genes that were not expressed in more than 70% of samples are deleted. The expressions of genes were log-transformed.

Moreover, we downloaded the gene expression profiles of 11 cancer types from ArrayExpress (E-MTAB-6690, pancreatic cancer; E-MTAB-6691, ovarian cancer; E-MTAB-6692, renal cancer; E-MTAB-6693, gastric cancer; E-MTAB-6694, prostate cancer; E-MTAB-6695, liver cancer; E-MTAB-6696, bladder cancer; E-MTAB-6697, melanoma cancer; E-MTAB-6698, colorectal cancer; E-MTAB-6699, lung cancer; and E-MTAB-6703, breast cancer). All the gene expressions were RMA normalized, merged, and batch effected via Combat method. Another six gene expression profiles and corresponding clinical information were downloaded from Gene Expression Omnibus and PubMed under the accession numbers GSE176307 (urothelial cancer), GSE28735 (pancreatic ductal adenocarcinoma), GSE72970 (colorectal cancer), GSE76019 (adrenocortical tumors), GSE78220 (melanomas), and previous studies [28, 29].

### Differential expression of TRIMs

Differential expression analysis was performed in 18 cancer types with five or more normal samples. Wilcoxon rank-sum test was used to evaluate the expression differences between normal and cancer samples. FDR method was used to adjust *p* values, and genes with FDR < 0.05 were considered as differentially expressed genes.

Moreover, Spearman Correlation Coefficient (SCC) between TRIM gene expressions was calculated by 'rcorr' function in the Hmisc R package. The difference of SCCs between the same subtypes and different subtypes was evaluated by Wilcoxon rank sum test.

### Calculation of TRIMs score

We utilized single-sample gene set enrichment analysis (ssGSEA) based on the gene set including TRIM genes to calculate TRIMs enrichment scores (TRIMs score) for each sample within the TCGA cohort. The ssGSEA algorithm firstly rank-normalized the gene expression value of a single sample $S$, and the genes are replaced by their ranks according their absolute expression $L = r_{1} ,r_{2} , \ldots , r_{N}$. The list is then ordered from the highest rank *N* to the lowest 1. Then, it used a sum (integration) of the difference between a weighted Empirical Cumulative Distribution Functions (ECDFs) of the TRIM gene signatures $G$ ($P_{G}^{w}$) and the ECDF of other genes NG ($P_{{{\text{NG}}}}$) to generate enrichment scores (ES):

$$\begin{aligned} P_{G}^{w} \left( {G,S,i} \right) & = \mathop \sum \limits_{{r_{j} \in G,j \le i}} \frac{{\left| {r_{j} } \right|^{\alpha } }}{{\mathop \sum \nolimits_{{r_{j} \in G}} \left| {r_{j} } \right|^{\alpha } }} ;\;P_{{{\text{NG}}}} \left( {G,S,i} \right) = \mathop \sum \limits_{{r_{j} \notin G,j \le i}} \frac{1}{{\left( {N - N_{G} } \right)}} \\ ES\left( {G,S} \right) & = \mathop \sum \limits_{i = 1}^{N} \left[ {P_{G}^{w} \left( {G,S,i} \right) - P_{{{\text{NG}}}} \left( {G,S,i} \right)} \right] \\ \end{aligned}$$

where the exponent of this quantity (*α*) is set to 1/4 to add a modest weight to the rank. This calculation is repeated for each signature and each sample in TCGA cohort.

We employed the ssGSEA algorithm via R packages (GSVA) [30] to comprehensively and systematically assess the potential functional impacts of TRIMs transcriptome alterations.

### Functional analysis of TRIMs

To identify the potential functional pathways associated with TRIM genes, we first calculated the SCCs between TRIMs scores and expressions of all genes. Genes were ranked by SCC and subjected into gene set enrichment analysis (GSEA) using the GSEA function in clusterProfiler package [31, 32]. The cancer hallmark pathways used in the analysis were downloaded from MSigDB database [33].

### Immune cell infiltrations in cancer

Immune cell infiltration levels of all TCGA samples calculated by CIBERSORT [34] were downloaded from TIMER2.0 [35]. SCC between TRIMs scores and the proportion of immune cell infiltration across cancer patients was calculated. The difference of immune infiltration levels between TRIMs scores-high group and low group was compared by Wilcoxon rank-sum test.

### TRIMs-based classification of cancer and normal samples

We used the ‘roc’ function in the pROC package [36] to evaluate the diagnostic accuracy of TRIMs scores in determining the presence of disease, with the classification data of normal or cancer and TRIMs score for each sample as input.

### Survival analysis

We downloaded the clinical survival information of all cancers from TCGA, and calculated the hazard ratio (HR) value of TRIMs score for each cancer using survival package and survminer package. Based on the ‘surv_cutpoint’ function, the survival information and TRIMs scores, we divided the samples into high and low groups. If HR > 1 and *p* < 0.05, we considered TRIMs score was a risky factor, and if HR < 1 and *p* < 0.05, we considered TRIMs score as a protective factor in cancer.

## Results

### Somatic mutations of TRIMs across cancer types

**Fig. 1:**
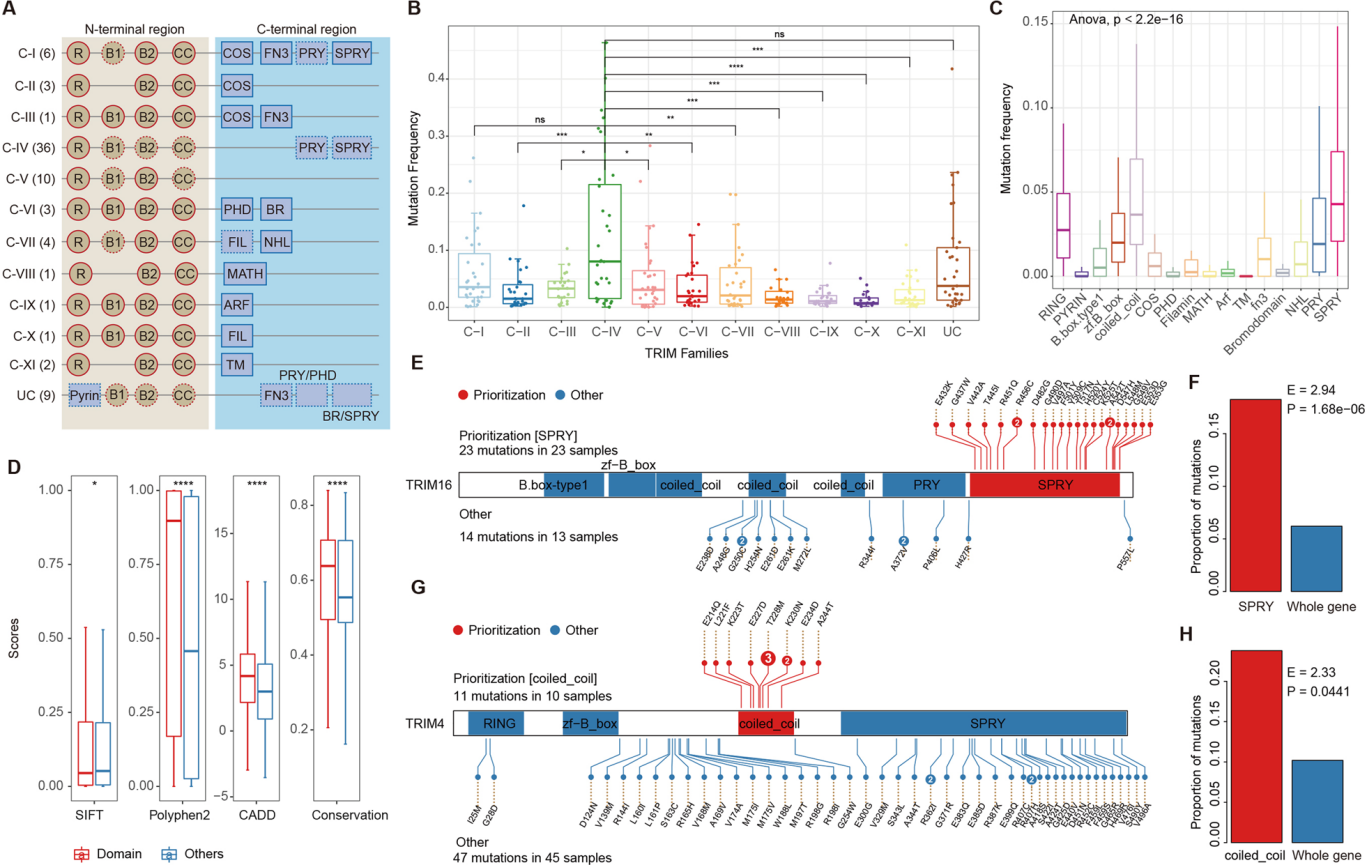
Genetic alterations of TRIM protein family. **A** Structure and classifications of TRIM family proteins (C-I to UC) are shown. Most TRIM proteins have a RING-finger domain (R), one or two B box zinc-finger domains (B1/B2) and a coiled-coil region (CC) in *N*-terminal region, and contain one or more carboxyl-terminal domains such as cos-box (COS), fibronectin type III repeat (FN3), PRY domain (PRY), SPRY domain (SPRY), PHD domain (PHD), bromodomain (BR), filamin-type I G domain (FIL), NCL1, HT2A and LIN41 domain (NHL), meprin and TRAF-homology domain (MATH), ADP-ribosylation factor family domain (ARF), and transmembrane region (TM). **B** Mutation frequency of TRIM proteins in different sub-classes (C-I to UC). Wilcoxon rank sum test, ****p < 0.0001, ***p < 0.001, **p < 0.01, *p < 0.05. **C** Mutation frequency in different domains of TRIM proteins. **D** Functional impact scores of mutations in domain versus other regions, evaluated by SIFT, Polyphen-2, CADD and conservation. Wilcoxon rank sum test, ****p < 0.0001, ***p < 0.001, **p < 0.01, *p < 0.05. **E** The somatic mutations and structure of TRIM16 are shown. Red, protein domains enriched somatic mutations. Blue, other domains. The number in the point represents the number of mutation samples. Mutation information includes mutation location and amino acids before and after mutation. **F** Proportion of mutations located in domain of interest and other regions for TRIM16. **G** The somatic mutations and structure of TRIM4. Red, protein domains enriched somatic mutations. Blue, other domains. The number in the point represents the number of mutation samples. Mutation information includes mutation location and amino acids before and after mutation. **H** Proportion of mutations located in domain of interest and other regions for TRIM4. ****p < 0.0001, ***p < 0.001, **p < 0.01, *p < 0.05

To comprehensively investigate the somatic mutations of TRIM genes in different cancer types, we obtained the somatic mutations in more than 10,000 patients from 33 cancers in TCGA. In total, we collected 77 TRIM genes, which were divided into 12 sub-classes according to the domain annotations (Fig. 1A). The majority of TRIM genes were classified into C-IV sub-class (Fig. 1A). We next calculated the mutation frequency of genes in different sub-classes and found that genes in C-IV exhibited a higher mutation frequency across cancer types, while genes in C-IX and C-X exhibited lower mutation frequency (Fig. 1B).

Protein domains are important regions for proteins to play their functions in various pathways [37]. We further calculated the mutation frequency of TRIM proteins in the domain levels. We found that the mutations in TRIM proteins mainly occurred in SPRY, coiled coil, and RING domains (Fig. 1C). In addition, we evaluated the functional impacts of somatic mutations by SIFT, Polyphen-2, CADD and conservation scores. We found that somatic mutations in the domains had significantly higher functional impact scores than mutations outside domains (Fig. 1D). These results suggested that cancer somatic mutations tend to occur inside protein domains and play deleterious functions in cancer.

We next prioritized protein domains enriched somatic mutations and identified seven genes (TRIM16, TRIM4, TRIM43, TRIM67, TRIM7, TRIM72 and TRIM73), as potential drivers (Fig. 1E–H and Additional file 1: Fig. S1). These genes were also predicted as potential driver genes by other computations methods [38–40]. Compared with mutations in other protein regions, TRIM16 and TRIM4 were significantly enriched mutation in SRRY and coiled-coil domains (Fig. 1E, G). There were significantly more mutations in SRRY domain of TRIM16, in which R456C and A542T were mutated in more than one cancer patient (Fig. 1E, F *E* = 2.94, *p* adjust = 1.68E−6). TRIM16 has been demonstrated to orchestrate the SQSTM1-KEAP1-NFE2L2 axis to mediate stress-induced biogenesis of protein aggregates, via interacting with NFE2L2 and SQSTM1 through the SPRY domain, and mediating the K63-link ubiquitination of NFE2L2 to enhance its stability [41]. In addition, mutations in TRIM4 were significantly enriched in the coiled-coil domain, with more than one patient having T228M and K230N mutations (Fig. 1G, H, *E* = 2.33, *p* adjust = 0.0441). Moreover, PRY was the domains enriching somatic mutations of TRIM7 and TRIM72 (Additional file 1: Fig. S1), and SPRY domain plays important roles in substrate recognition [12, 42]. Together, these results suggested there were widespread somatic mutations in TRIM gene family and cancer mutations were significantly enriched in protein domains.

### Widespread transcriptome alterations of TRIMs in cancer

**Fig. 2:**
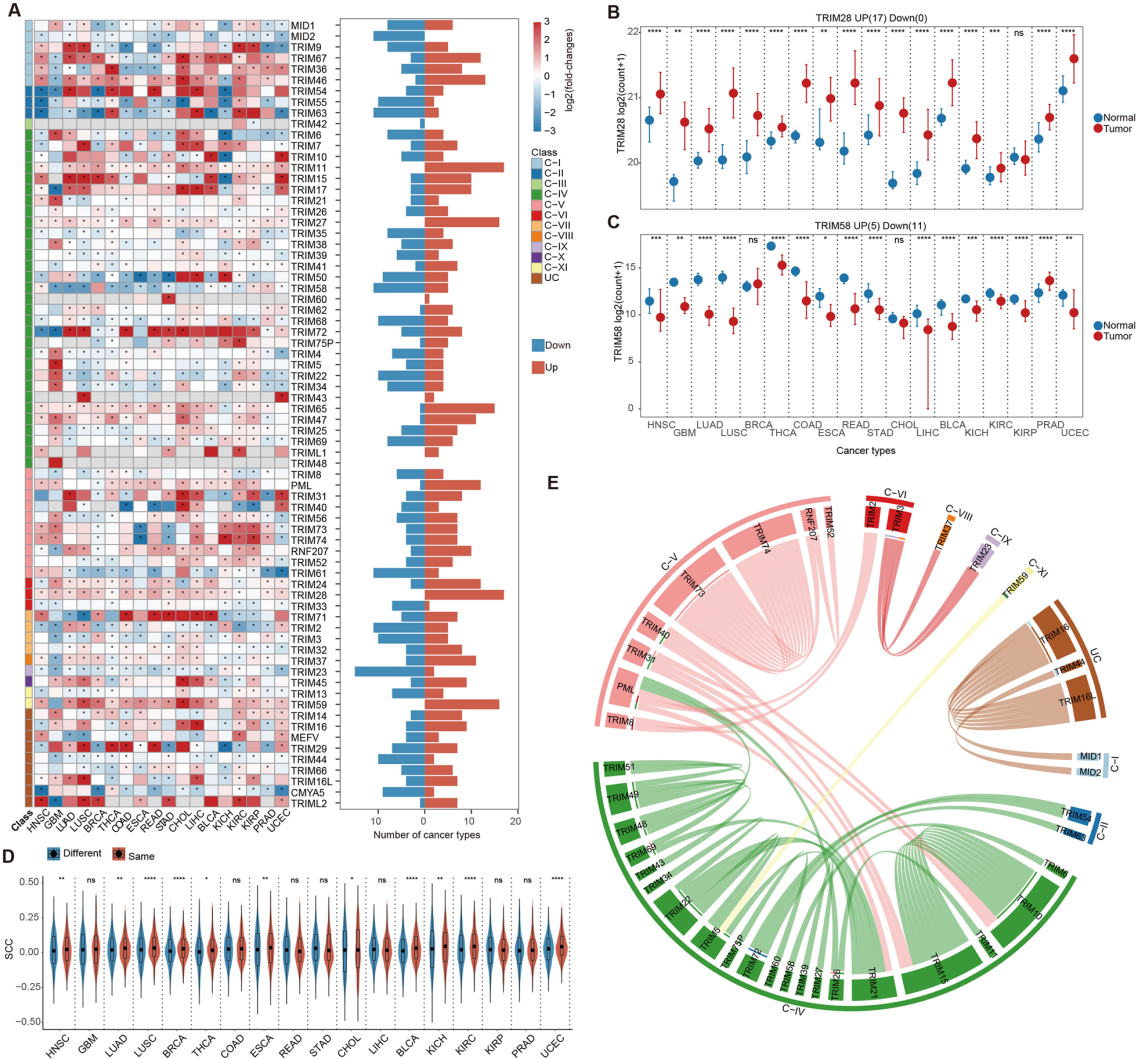
Perturbations of the expression of TRIM protein family in cancer. **A** Landscape of differential expression of TRIM proteins family between normal and tumor samples across cancer types. Heatmap on the left panel showing the log2(fold-changes) of TRIM genes across cancer types, where '*' indicates significant difference. Bar plots on the right panel showing the number of cancers that the corresponding TRIM genes perturbed. Red represents up-regulated expression in cancer, and blue represents decreased expression. **B** and **C** Box plots showing the expression levels of TRIM28 (**B**) and TRIM58 (**C**) between normal and tumor samples across 18 cancer types. The point represents the median value of expression, and sides of the line represent the upper and lower quartiles of that. Wilcoxon rank sum test, ****p < 0.0001, ***p < 0.001, **p < 0.01, *p < 0.05. **D** Boxplots showing the SCC distributions of TRIM genes within and between different sub-classes. Red represents Spearman correlation coefficient between the same sub-class, and blue represents that between different sub-classes. Wilcoxon rank sum test, ****p < 0.0001, ***p < 0.001, **p < 0.01, *p < 0.05. **E** Circos plot showing the co-expression between TRIM genes. Gene pairs with *p* value < 0.05 and SCC > 0.75 was plotted. One line indicates strong correlation in one cancer, and multiple lines between two genes indicate strong correlation in multiple cancers

To investigate the expression pattern of the TRIM family proteins in cancer, we analyzed the gene expression profile of 18 cancers with 5 or more normal samples. In total, 72 TRIM genes with non-zero expression in at least 30% of samples in each cancer were analyzed. We found that 71/72 (98.61%) of the TRIM genes were differentially expressed in at least one cancer (Fig. 2A, FDR < 0.05). Several TRIM genes exhibited similar expression patterns in various cancer types, such as TRIM28 and TRIM59, which were extensively highly expressed in cancer (Fig. 2B and Additional file 1: Fig. S2A). In addition, TRIM58 or TRIM23 exhibited lower expression in multiple cancer types (Fig. 2C and Additional file 1: Fig. S2B). TRIM28 was significantly overexpressed in 17 cancer types (Fig. 2B), which was consistent with the observations that TRIM28 was more prone to promote tumorigenesis [43, 44]. TRIM28 also played a role in the development of cancer by reducing autophagy or inhibiting anti-tumor immunity and immune checkpoint blockade [45, 46]. In contrast, TRIM58 was ubiquitously low expressed in 11 cancers (Fig. 2C). Notably, the expression levels of TRIM58 were negatively correlated with methylation levels in various cancers except KIRC (Additional file 1: Fig. S3A–E). Almost all TRIM58 methylation subtypes are significantly negatively correlated with their expression levels in various cancers, such as LIHC and COAD patients (Additional file 1: Fig. S3B, C). In addition, we further analyzed the relationship between methylation of TRIM58 and survival outcomes of patients using a web tool MethSurv (Additional file 1: Fig. S3F) [47]. It suggested that renal cancer-related patients with higher methylation level of TRIM58 are associated with poor survival (Additional file 1: Fig. S3G, H, ACC, cg20146541, HR = 4.204; KIRP, cg26157385, HR = 2.423). It has been demonstrated that abnormal methylation of TRIM58 may lead to down-regulation of its expression, which leads to increased aggressiveness of cancer cells and reduced survival rate of cancer patients [48–50]. Recent studies have highlighted the possible role of TRIM58 as a tumor suppressor gene [50], which degrades β-catenin through ubiquitination, resulting in the inactivation of β-catenin signaling. There results suggested the widespread transcriptome alterations of TRIM genes in cancer. Furthermore, additional cohorts were used to analyze the gene expression of TRIMs in 11 independent cancer types. We found that 67 TRIM genes exhibited differential expression in at least one cancer type (Additional file 1: Fig. S4A). In particular, TRIM28 exhibited upregulation in seven cancer types, and immunohistochemical results from The Human Protein Atlas (HPA) database [51] also showed that TRIM28 has higher expression in liver related cancer tissues than in normal liver tissues. (Additional file 1: Fig. S4B, D). Consistent with previous results, TRIM58 was also down-regulated in various cancers, and the expression of its coding protein in liver tumor tissues is reduced (Additional file 1: Fig. S4C, D). It indicated that TRIM28 was an oncogene, while TRIM58 is more likely to be a tumor suppressor gene. These results further confirmed the importance of TRIM gene in tumorigenesis.

In addition, genes do not function in isolation, and evidence has shown that collaboration among genes exists in the context of cancer [52]. We found that several TRIM genes exhibited similar expression patterns in various cancers. Therefore, we calculated SCCs between TRIM genes, and found that TRIM genes belonging to the same sub-classes exhibited significantly higher SCCs in the majority of cancer types (Fig. 2D). In particular, TRIM genes from the same sub-classes were significantly positive correlation (Fig. 2E), when we considering the significantly correlated TRIM gene pairs (*p* adjust < 0.05 and *R* > 0.75). Since TRIM family sub-classes were classified according to the annotations of functional domains, members from the same sub-classes with similar domains may play roles in cancer pathways coordinately. All these results indicated that TRIM genes were dysregulated in expression across cancer types, and genes in the same sub-classes tend to be co-expressed in cancer.

### TRIMs regulate cancer immunology pathways

**Fig. 3:**
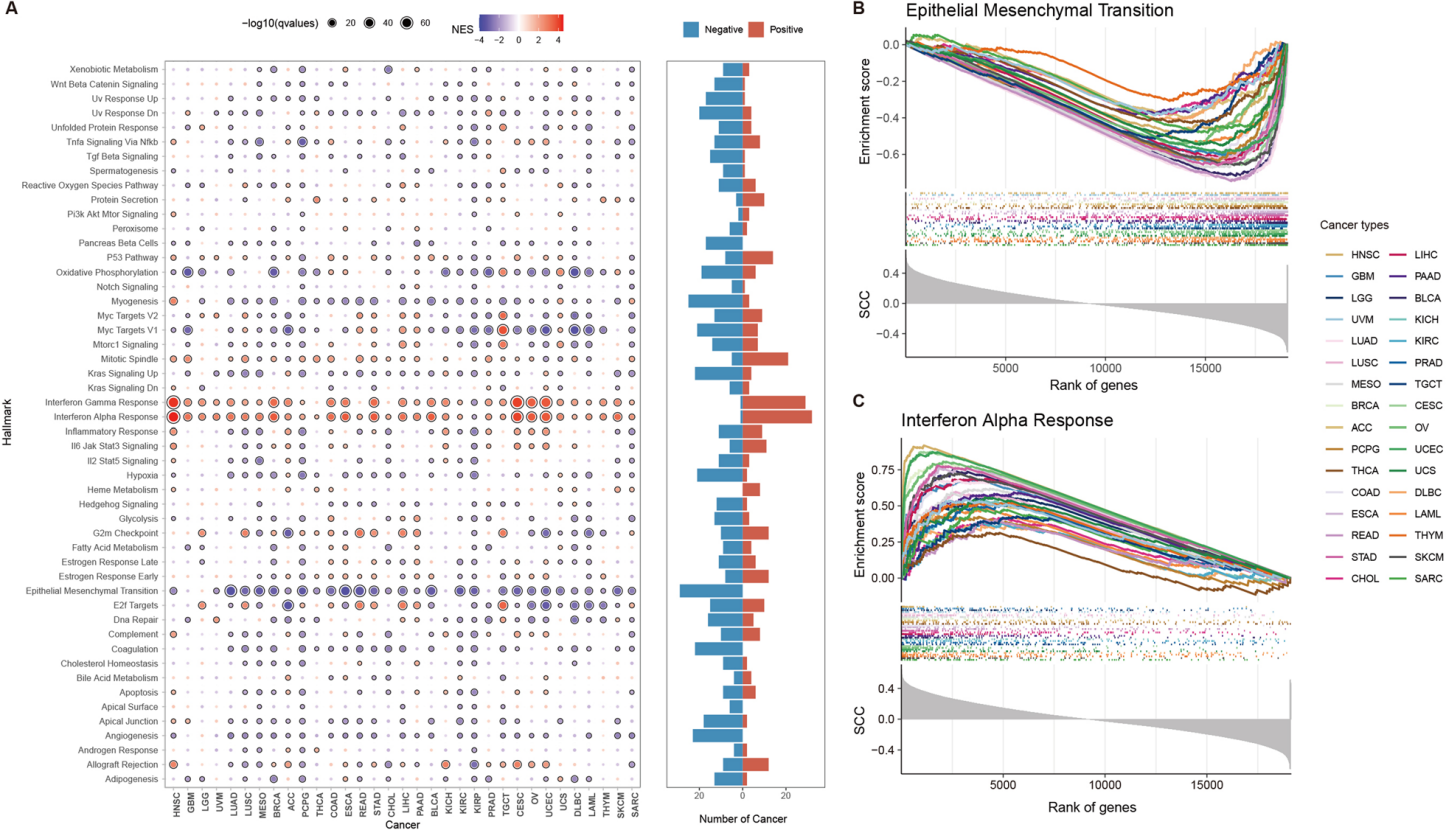
Functional pathways of TRIM protein family across cancer types. **A** Gene set enrichment analysis (GSEA) of TRIM genes in various cancers. Heat map showing the normalized enrichment scores (NES) and size of the dots corresponding to the − log10(adjusted *p* values). Red indicates active pathway, while blue indicates inhibitory pathway. The black border demonstrates significant results. Bar plots on the right panel showing the number of cancers that the corresponding pathway enriched (red) or depleted (blue). **B** and **C** The enrichment score (ES) distribution for the genes positively or negatively co-expressed with TRIMs scores in EMT pathway (**B**) and interferon alpha response pathway (**C**). Each line is for one cancer and lines are colored by cancer types

TO systematically explore the potential functional impacts of TRIMs transcriptome alterations in cancer, we first calculated a TRIMs score for each cancer patient based on single-sample gene set enrichment analysis (ssGSEA) algorithm [32]. We next evaluated the correlation between TRIMs scores and expressions of all protein coding genes. Protein coding genes were ranked by the correlation and subjected into gene set enrichment analysis (GSEA). The hallmark gene sets were evaluated across cancer types. We found that TRIMs scores are significantly correlated with cancer- or immune-related pathways across cancer types (Fig. 3A, adjusted *p* < 0.05), such as epithelial-mesenchymal transition (EMT) and interferon response pathways. In particular, EMT and Kras signaling up pathways were negatively correlated with TRIMs scores across cancer types (Fig. 3A). Interferon alpha response and interferon gamma response pathways exhibited consistently positively correlated with TRIMs scores across cancer types (Fig. 3A).

EMT is a cellular program, which can enhance tumor-initiating and metastatic potential, and contribute to the development of malignant tumors [53]. Interferons (IFNs) are a family of cytokines that protect against complex diseases by activating immune responses, and of great significance for preventing and treating cancer [54]. TRIMs scores were significantly negatively correlated EMT pathway in 29 cancer types, and positively correlated with the activation of interferon alpha response related pathways in 32 cancer types (Fig. 3B, C). These results were consistent with previous observations that many members of the TRIM family were induced by IFN responses, and played an important role in IFN-mediated innate immune regulation [13, 55, 56]. In addition, we found that TRIMs scores were correlated with E2F targets and G2M checkpoint pathways in several cancer types (Additional file 1: Fig. S5A, B). We further divided the cancer samples into two groups with high or low TRIMs scores in each cancer, and identified the differentially expressed genes (DEGs) between two groups. Functional enrichment analysis revealed that up-regulated genes were significantly enriched in immune-mediated, antigen presentation and response-related functions (Additional file 1: Fig. S5C). Taken together, all these results suggested that perturbations of TRIMs play important roles in immune- and cancer-related pathways.

### TRIMs correlated with immune checkpoints and immune cell infiltrations

**Fig. 4:**
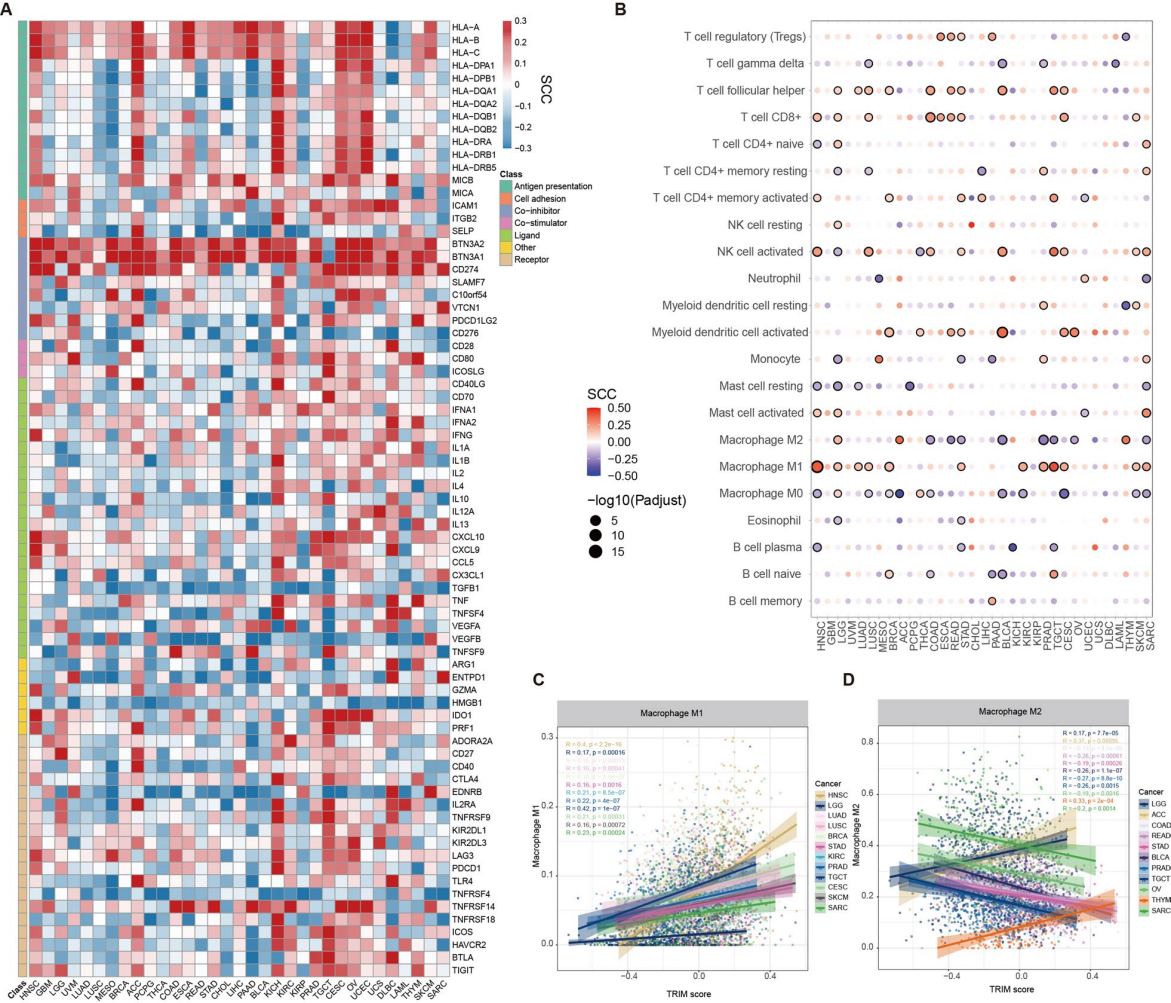
Immune regulation of TRIM protein family across cancer types. **A** The landscape of correlations between TRIMs scores and expression of immune regulatory factors which divided into seven categories: antigen presentation, cell adhesion, co-inhibition, co-stimulation, receptor, ligand and others is shown. Heat map showing Spearman correlation coefficient (SCC) between TRIMs scores and expression of immune regulatory factors. Red indicates positive correlation, blue indicates negative correlation. Red indicates positive correlation, while blue indicates negative. **B** The correlations between TRIMs scores and immune cell infiltrations across cancer types. Dot plot showing Spearman correlation coefficient (SCC) and size of the dots corresponding to the − log10(adjusted *p* values). Red indicates positive, while blue indicates negative. The black border demonstrates significant correlation. **C** and **D** Scatter plots showing the correlations between TRIMs scores and Macrophage M1 (**C**) or M2 (**D**) infiltrations across cancer types. Dot and lines are colored by cancer types

To further determine the correlation between TRIMs scores and immunity in the tumor microenvironment, we explored the correlation with 78 immune regulatory factors, which were mainly divided into seven categories: antigen presentation, cell adhesion, co-inhibition, co-stimulation, receptor, ligand and others [57]. We found that TRIMs scores were significantly positively correlated with immune regulatory factors in the majority of cancers (Fig. 4A), such as MHC class I member HLA-A/B/C, BTN3A family activating T cells, interferon related factors (IFNA1, IFNG), TNFRSF14, which is a member of the pro-inflammatory tumor necrosis factor superfamily (TNF) and oncogene CD274 (PD-L1) (Additional file 1: Fig. S6A). There were higher numbers of correlations observed in gynecological cancers, such as CESC, OV and UCEC (Additional file 1: Fig. S6B).

We further explored the association between TRIMs scores and 22 immune cell infiltrations. We found that TRIMs scores were significantly correlated with immune cells in cancer (Fig. 4B and Additional file 1: Fig. S6C), in particular for T cell follicular helper, CD8+ T cell, activated NK cell, Macrophage M1 and M2 (Fig. 4B). TRIMs scores were significantly positively correlated with pro-inflammatory Macrophage M1 and negatively correlated with anti-inflammatory Macrophage M2 in numerous cancer types (Fig. 4C, D). Moreover, TRIMs scores were significantly correlated with T cell follicular helper and activated NK cell infiltrations in more than ten cancer types (Additional file 1: Fig. S6D, E). Next, we compared the immune cell infiltration levels between patients with low or high TRIMs scores. We found that there were great differences in the proportion of immune cell infiltrations between two groups at the pan-cancer level (Additional file 1: Fig. S6F). For T cell follicular helper, CD8+ T cell, T cell regulatory (Tregs), activated NK cell, Macrophage M1 and Mast cell activated, patients in TRIMs scores high group had a significantly higher proportion of immune cell infiltrations (Additional file 1: Fig. S6F).

### Clinical survival associations of TRIMs with cancer

**Fig. 5:**
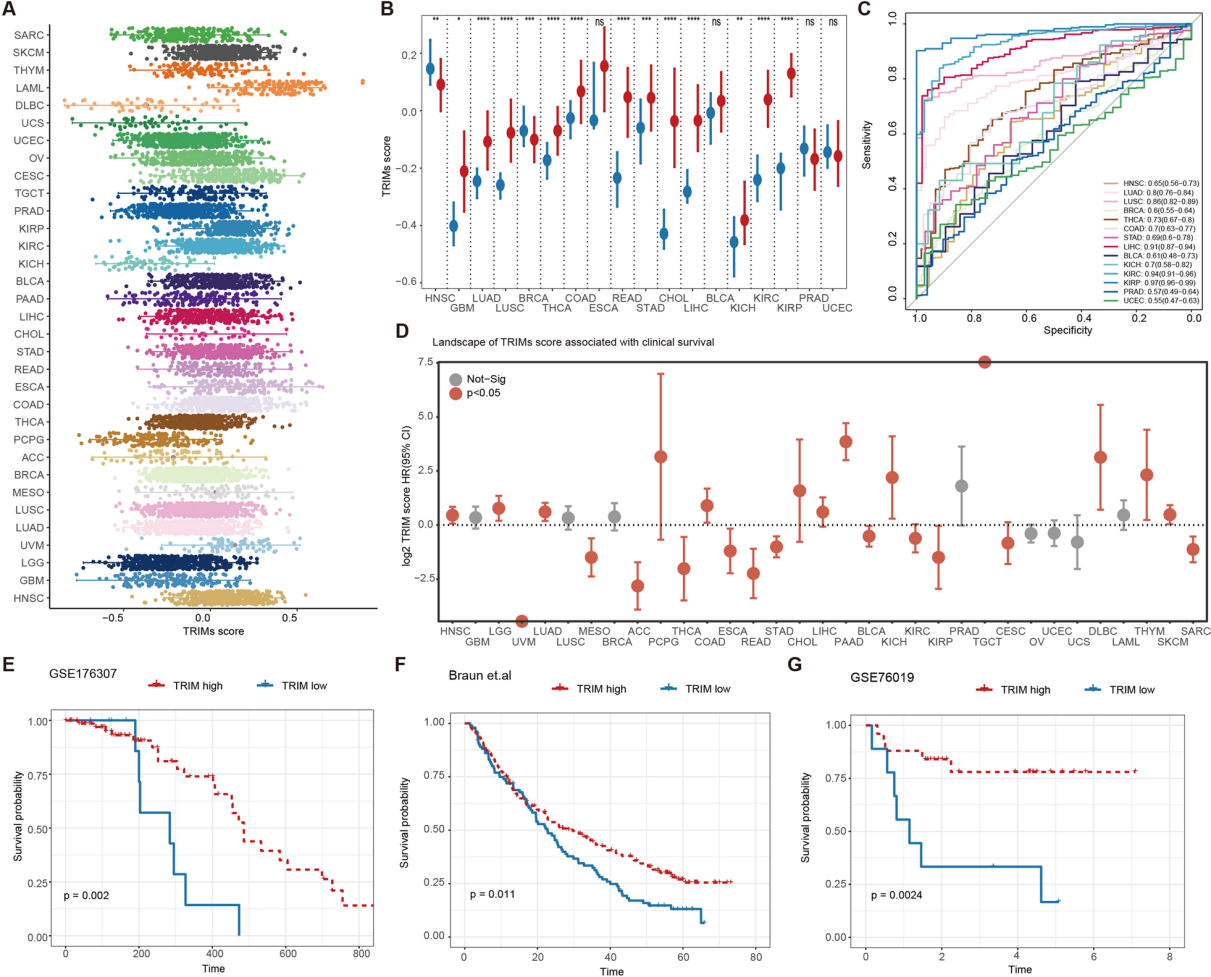
Clinical associations of TRIMs score across cancer types. **A** The distributions of TRIMs score across cancer types. Dot and lines are colored by cancer types. Within each cancer, the scattered dots represented TRIMs score values. **B** Boxplots showing the distributions of TRIMs scores between normal (blue) and tumor (red) samples. The point represents the median value of TRIMs score in each cancer, and sides of the line represent the 25th and 75th quartiles of that. The statistical difference of two group was compared through the Wilcoxon rank sum test, ****p < 0.0001, ***p < 0.001, **p < 0.01, *p < 0.05. **C** Area under the ROC curves (AUCs) for classifiers based on TRIMs scores in TCGA cohort. Legends show the basic information of each cancer. Lines are colored by 14 cancer types with 15 or more normal samples. '*h*(*m*–*n*)' indicates the HR values and 95%CI. **D** The distribution of hazard ratios (HR) based on TRIMs scores across different cancer types. The point represents the HR value, and sides of the line represent 95%CI. Red dots and lines indicate significant result. **E**–**G** Kaplan–Meier survival plot of patients grouped by high versus low TRIMs scores. The group with low TRIMs scores (blue) has poorest survival, whereas the high TRIMs score group (red) is associated with better outcomes (log-rank test *p* value < 0.05)

Considering the associations between TRIMs scores and immunity in tumor microenvironment, we next evaluated the impact of TRIMs scores on clinical survival of cancer patients. We found there were a wide range of TRIMs scores for patients within the same cancer and across cancer types (Fig. 5A, ANOVA, *p* value < 2.2E−16), suggesting the great heterogeneities in cancer. Patients in acute myeloid leukemia (LAML) have the highest levels of TRIMs scores on average across all cancers, whereas patients in lymphoid neoplasm diffuse large B-cell lymphoma (DLBC) have the lowest TRIMs scores (Fig. 5A). Next, we compared the TRIMs scores between tumor and normal samples in 18 cancers with five or more normal samples. We found that there were significant differences in TRIMs score between normal and tumor samples in 14 cancers (Fig. 5B). TRIMs scores in cancer samples were significantly higher than those in normal samples across cancer types (Fig. 5B). We also explored the proportion of samples with high or low TRIMs scores in each cancer, and the results showed that there were more samples in the high TRIMs scores group (Additional file 1: Fig. S7).

To further evaluate to what extent the TRIMs scores in distinct the cancer and normal patients, we used pROC package to calculate the area under the receiver operating characteristic (ROC) curve (AUC), sensitivity, and specificity. We selected 14 cancers with 15 or more normal samples for further analysis. We found that TRIMs scores can accurately distinguish normal and cancer samples in the majority of cancer types (Fig. 5C). TRIMs score achieved better accuracy in kidney related cancer (AUC = 0.94 and 0.97 in KIRC and KIRP), liver related cancer (AUC = 0.91) and lung related disease (AUC = 0.8 and 0.86 in LUAD and LUSC) (Fig. 5C). These results were consistent with the observations that TRIM genes expression greatly altered in cancer.

We also evaluated whether TRIMs scores could predict survival of patients. We found that TRIMs scores were significantly associated with overall survival in 25/33 (76%) cancer types (Fig. 5D, *p* value < 0.05). We also obtained other cancer cohorts annotated with clinical information from Gene Expression Omnibus (GEO) and Published literature [29, 58–61]. Univariable Cox-regression analyses revealed a cancer-specific association of TRIMs scores in predicting survival times of patients. TRIMs scores were a favorable prognosis factor and patients with higher TRIMs scores were with better survival in metastatic urothelial cancer (Fig. 5E and Additional file 1: Fig. S8A, log-rank *p* = 0.002 and HR = 0.319), advanced clear cell renal cell carcinoma (Fig. 5F, log-rank *p* = 0.011 and HR = 0.697) and adrenocortical (Fig. 5G, log-rank *p* = 0.0024 and HR = 0.203). However, patients with higher TRIMs score were associated with lower survival rates in colorectal cancer and pancreatic cancer (Additional file 1: Fig. S8A). It is consistent with previous results. All these results suggested that TRIMs scores can effectively predict the clinical outcome of many cancer patients.

### Potential therapeutic response associated with TRIMs in cancer

**Fig. 6:**
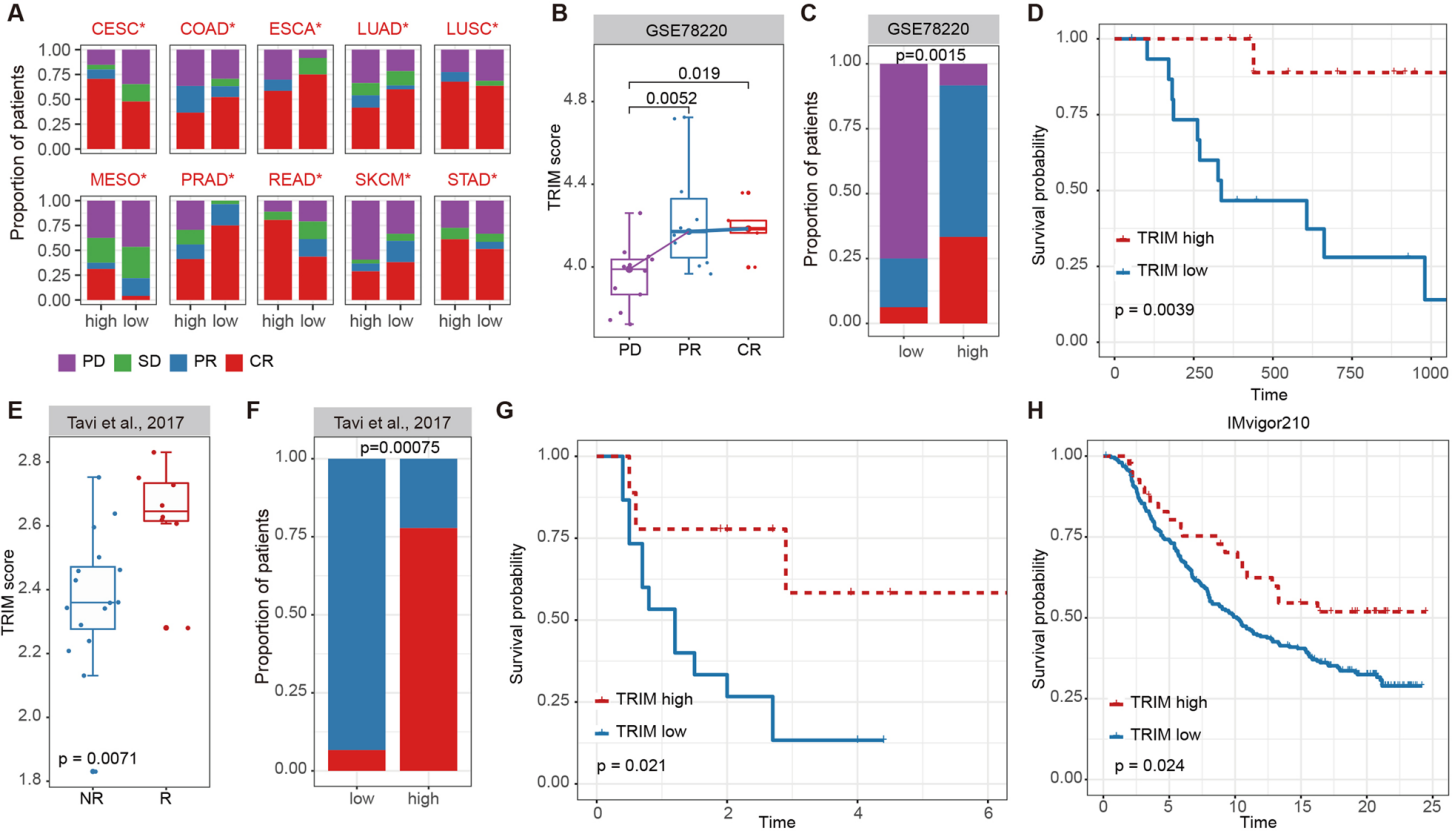
Clinical therapeutic response associated with TRIMs in cancer. **A** The proportions of patients for therapeutic response between TRIMs scores high and low groups in TCGA cohorts. Patients were classified into progressive disease (PD), stable disease (SD), partial response (PR) and complete response (CR) based on drug treatment responses. Fisher’s exact test was used to compare statistical difference of two group. **B** Boxplots showing the distributions of TRIMs scores for patients with different therapeutic response to anti-PD1 immunotherapy. The statistical difference of three response stages was compared through the Wilcoxon rank sum test. Patients with better responses have higher TRIMs scores (*p* value < 0.05). **C** Proportions of patients with different therapeutic response to anti-PD1 immunotherapy in TIRMs scores high versus low groups. Fisher’s exact test was used to compare statistical difference of two group (*p* value = 0.0015). **D** Kaplan–Meier survival plot of patients grouped by high versus low TRIMs scores in anti-PD1 immunotherapy cohort (GSE78220). The group with low TRIMs scores (blue) has poorest survival, whereas the high TRIMs score group (red) is associated with better outcomes (log-rank test *p* value = 0.0039). **E** Boxplots showing the distributions of TRIMs scores for patients with different therapeutic response to anti-CTLA-4 immunotherapy. The statistical difference of three response stages was compared through the Wilcoxon rank sum test (*p* value = 0.0071). **F** Proportions of patients with different therapeutic response to anti-CTLA-4 immunotherapy in TIRMs scores high versus low groups (Fisher’s exact test, *p* value = 0.00075). **G** Kaplan–Meier survival plot of patients grouped by high versus low TRIMs scores in anti-CTLA-4 immunotherapy cohort. The group with low TRIMs scores (blue) has poorest survival, whereas the high TRIMs score group (red) is associated with better outcomes (log-rank test *p* value = 0.021). **H**. Kaplan–Meier survival plot of patients grouped by high versus low TRIMs scores in IMvigor210 cohorts. The group with low TRIMs scores (blue) has poorest survival, whereas the high TRIMs score group (red) is associated with better outcomes (log-rank test *p* value = 0.024)

TRIM proteins play important roles in the regulation of biological behaviors of cancer cells. Next, we further explored the potential value of TRIMs scores in cancer treatment. We first analyzed the drug therapy response of cancer patients based on TRIMs scores. Patients were classified into progressive disease (PD), stable disease (SD), partial response (PR) and complete response (CR) based on drug treatment responses. We found that there were significant differences in treatment response between TRIMs scores high and low groups in ten cancer types (Fig. 6A). In particular, patients with high TRIMs scores had a higher proportion of better treatment response in COAD, ESCA, LUAD, PRAD and SKCM (Fig. 6A, Fisher’s exact test, *p* value < 0.05).

In addition, we collected other cancer cohorts with drug therapy from literature. In melanoma patients treated with anti-PD-1 checkpoint inhibitors [60], we found patients response to treatment (CR and PR) were with higher TRIMs scores (Fig. 6B, *p* = 0.019 and 0.0052). Moreover, a higher proportion of patients in high TRIMs scores group were likely to response to drug treatment (CR and PR) (Fig. 6C, *p* = 0.0015, Fisher’s exact test). In addition, patients with higher TRIMs scores were with better survival (Fig. 6D, log-rank *p* = 0.0039). Moreover, for melanoma patients treated with CTLA-4 blockade [28], we found that TRIMs scores of patients response or not response to drug treatment were significantly different (Fig. 6E, *p* = 0.0071). The proportion of patients that response to treatment was higher in the high TRIMs scores group (Fig. 6F, *p* = 0.00075, Fisher’s exact test). Moreover, patients with high TRIMs scores had better survival (Fig. 6G, log-rank *p* = 0.021). TRIMs scores can relatively accurately predict the treatment effects with anti-PD-1 checkpoint inhibitor or CTLA-4 blockade in melanoma (Additional file 1: Fig. S8B, AUC = 0.84 and 0.82). In the urothelial carcinoma treated with immunotherapy, we found that patients with high TRIMs scores had a better survival than those with low TRIMs scores (Fig. 6H, log-rank *p* = 0.024), which was consistent with previous results. In summary, TRIMs scores may be able to accurately predict drug response and clinical survival for some cancer types.

## Discussion

Emerging evidence has revealed that TRIM proteins as regulators of cancer growth and immune-related pathways. However, the comprehensive genetic and transcriptome landscape of TRIM genes across cancer types remain unclear, suggesting the need for comprehensive review analysis. In this study, we performed comprehensive review and analyses of the somatic mutations, transcriptome dysregulation, and clinical relevance of TRIM genes across cancer types. We found that somatic mutations were likely to be observed in the domain regions of TRIM proteins. We prioritized several TRIM genes with enrichment of mutations in protein domain, such as TRIM16, TRIM4 and TRIM7. These genes provided candidates for further functional validation in cell lines or animal models.

Moreover, the transcriptome of TRIM genes exhibited greatly perturbed in cancer. TRIM11, TRIM27, TRIM28 and TRIM59 have higher expression in almost all cancers. Previous studies have shown that overexpression of these genes promotes the proliferation, migration and invasion of many cancers through a variety of mechanisms, such as beta-catenin signaling, AKT signaling, P53 activity, and epithelial mesenchymal transition. On the contrary, TRIM23, TRIM61 and TRIM58 have lower expression in cancer. It has been reported that TRIM58 may inhibit tumor growth through interaction with pyruvate kinase M2 or beta-catenin signaling [62]. In addition, we found that methylation and expression of TRIM58 were significantly negatively correlated, and patients with hypermethylation had poor survival in renal-related cancers. Immunohistochemical results further demonstrate the role of TRIM28 and TRIM58 in cancer. Perturbation of TRIM genes affected numerous cancer-related pathways. We proposed the TRIMs scores index for further investigating the functional pathways regulated by TRIM genes. We found that EMT, interferon alpha response and interferon gamma response were consistently correlated with TRIMs scores across cancer types. Emerging evidences suggest that TRIM family play important roles in these pathways. For example, TRIM50 can suppress pancreatic cancer progression and reverse EMT via Snail1, which is a key regulator of EMT [63]. TRIM11 can also promote proliferation, migration and EMT by activating the beta-catenin signaling in cancer [64]. In addition, TRIMs scores were correlated with E2F targets and G2M checkpoint pathways, both of which are related to cell cycle. Several studies have shown that TRIM11 and TRIM59 may promote proliferation and metastasis by regulating cyclin, and TRIM16 also plays a role in the process of cell cycle by changing the expression of cyclin D1 and p27. These results suggested that TRIM proteins play important roles in cancer immunology.

TRIM proteins have been intensively studied as essential modulators in immune responses. We also found that TRIMs scores were significantly correlated with the expression of immune checkpoints and immune cell infiltration levels in cancer. TRIMs scores were positively correlated with M1 macrophage infiltrations and negatively correlated with M2 macrophage infiltrations. Both M1 macrophages and M2 macrophages have been demonstrated to be closely related to inflammatory responses, among which M1 macrophages are mainly involved in pro-inflammatory responses and M2 macrophages are mainly involved in anti-inflammatory responses [65]. In addition, TRIMs scores were significantly associated with T cell follicular helper cells, CD8^+^ T cell and activated NK cell infiltration, which are closely related to immune response. These observations revealed that TRIM proteins play important roles in inflammatory and immune responses.

Well-established ubiquitylation mechanisms are understood on some key proteins (such as P53, NF-kb, and PI3K/AKT) of some signaling pathways. TRIM proteins have also been found to be targeted by several drugs. For example, target therapy of TRIM14 can inhibit osteosarcoma aggressiveness through the NF-kb signaling pathway [66]. We found that TRIMs scores can effectively distinct the normal and cancer patients. TRIMs scores are also associated with clinical survival and drug treatment responses, in particular immune therapies. All these results suggested that TRIM family members are potential biomarkers for cancer diagnosis and prognosis, and potential therapeutic targets in cancer. However, despite the tremendous progress that has been made in understanding the functions and signaling pathways of TRIM proteins responsible for tumor, limited TRIM protein-based therapeutics for cancers are approved by the U.S. Food and Drug Administration (FDA) or entering clinical trials.

Our analysis of TRIMs genes was performed by cancer type separately. Because of the great heterogeneity between cancers, the results in each cancer are different. For example, in the survival analysis, TRIMs scores were significantly associated with overall survival in 25 cancer types. In 12 of these cancers, patients with high TRIMs scores had better survival, while in the remaining 13 cancers, patients with high TRIMs scores were with poorer survival (Fig. 5D). The additional cohort obtained similar results. In investigating response to treatment, results from the TCGA cohort showed that TRIMs scores were positively associated with treatment response when as a protective factor in cancer, and negatively associated with treatment response when as a risk factor in cancer.

## Conclusions

In summary, genome-wide analysis of the somatic mutations and transcriptome supported the important roles of TRIM proteins in tumorigenesis. This study provided a comprehensive review and analyses of genetic and pharmacogenomics landscape of TRIM proteins across cancer types, which will shed light on the future development of therapeutic targets.

## Supplementary Information

**Additional file 1: Fig. S4.** Mutations of TRIM genes across cancer types. **Fig. S2.** Perturbations of the expression of TRIM protein family in cancer. **Fig. S3.** Methylation of TRIM58 in various cancers. **Fig. S4.** Perturbations of the expression of TRIM protein family in additional cohorts. **Fig. S5.** Functional pathways of TRIM protein family across cancer types. **Fig. S6.** Immune regulation of TRIM protein family across cancer types. **Fig. S7.** Proportion of patients with high or low TRIMs scores across cancer types (Red, TRIMs score high group; Blue, TRIMs score low group). **Fig. S8.** Clinical associations of TRIMs scores in additional cohorts.

## Data Availability

All data generated or analyzed during this study are included in this published article. The gene expression profiles and clinical data can be found at the GDC portal and GEO (https://www.ncbi.nlm.nih.gov/geo/). Software and resources used for the analyses are described in each method section and can be accessed from GitHub (https://github.com/ComputationalEpigeneticsLab/Pan-cancer-Analysis-of-Tripartite-Motif).
