# Supplementary material for: Pan-cancer illumination of TRIM gene family reveals immunology regulation and potential therapeutic implications
Source: Hum Genomics. 2022 Dec 2;16:65. doi: 10.1186/s40246-022-00441-9 (PMC9719184; doi:10.1186/s40246-022-00441-9)
Supplement: Supplementary file 1 — Additional file 1: Fig. S4. Mutations of TRIM genes across cancer types. Fig. S2. Perturbations of the expression of TRIM protein family in cancer. Fig. S3. Methylation of TRIM58 in various cancers. Fig. S4. Perturbations of the expression of TRIM protein family in additional cohorts. Fig. S5. Functional pathways of TRIM protein family across cancer types. Fig. S6. Immune regulation of TRIM protein family across cancer types. Fig. S7. Proportion of patients with high or low TRIMs scores across cancer types (Red, TRIMs score high group; Blue, TRIMs score low group). Fig. S8. Clinical associations of TRIMs scores in additional cohorts. [file 40246_2022_441_MOESM1_ESM.docx]

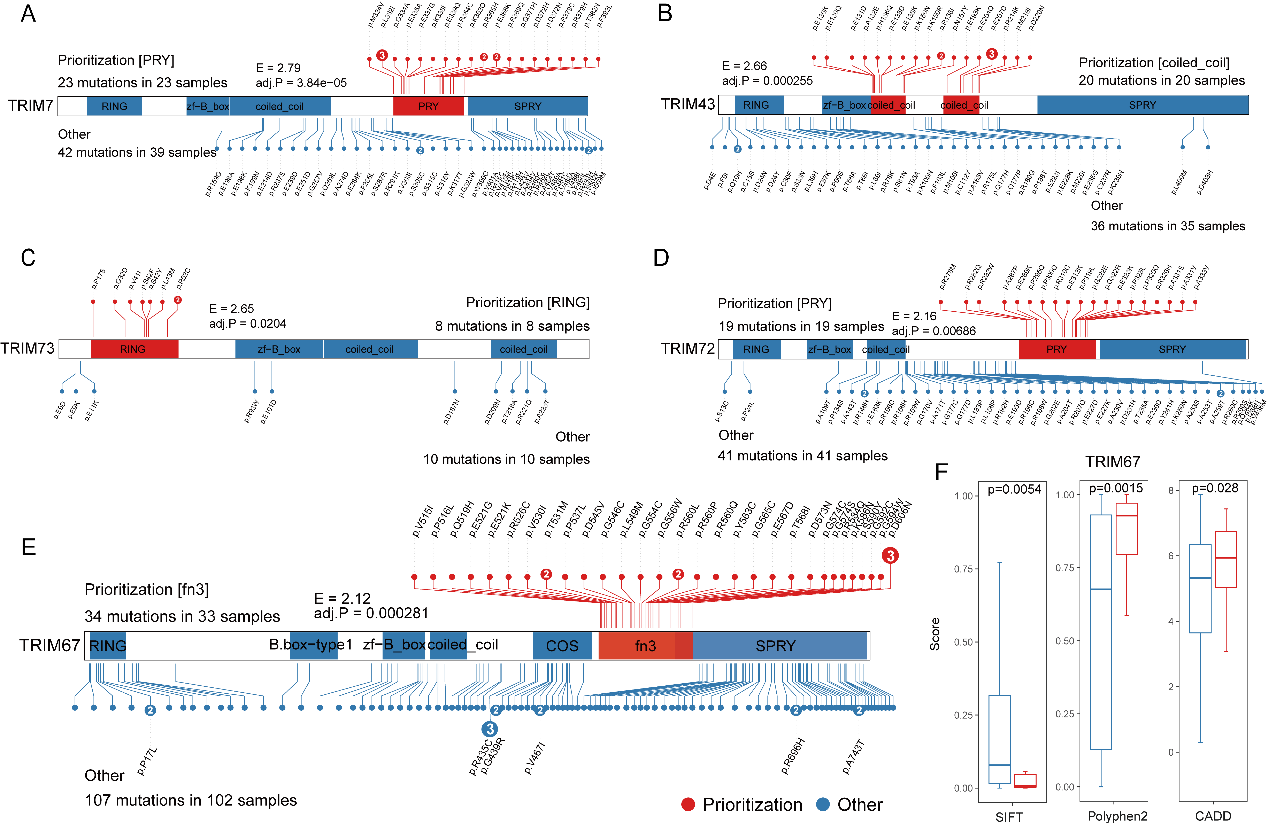


**Supplementary figures. Fig. S1. Mutations of TRIM genes across cancer types.** (A-E) The somatic mutations and structure of TRIM7 (A), TRIM43 (B), TRIM73 (C), TRIM72 (D) and TRIM67 (E). Red, protein domains enriched somatic mutations. Blue, other domains. The number in the point represents the number of mutation samples. Mutation information includes mutation location and amino acids before and after mutation. (F) Boxplots showing the functional impact scores for mutations in prioritization vs. other domain of TRIM67, evaluated by SIFT, Polyphen-2, CADD and conservation (Wilcoxon rank sum test). Mutations in the prioritized domain are more harmful than those in other regions (p value < 0.05).


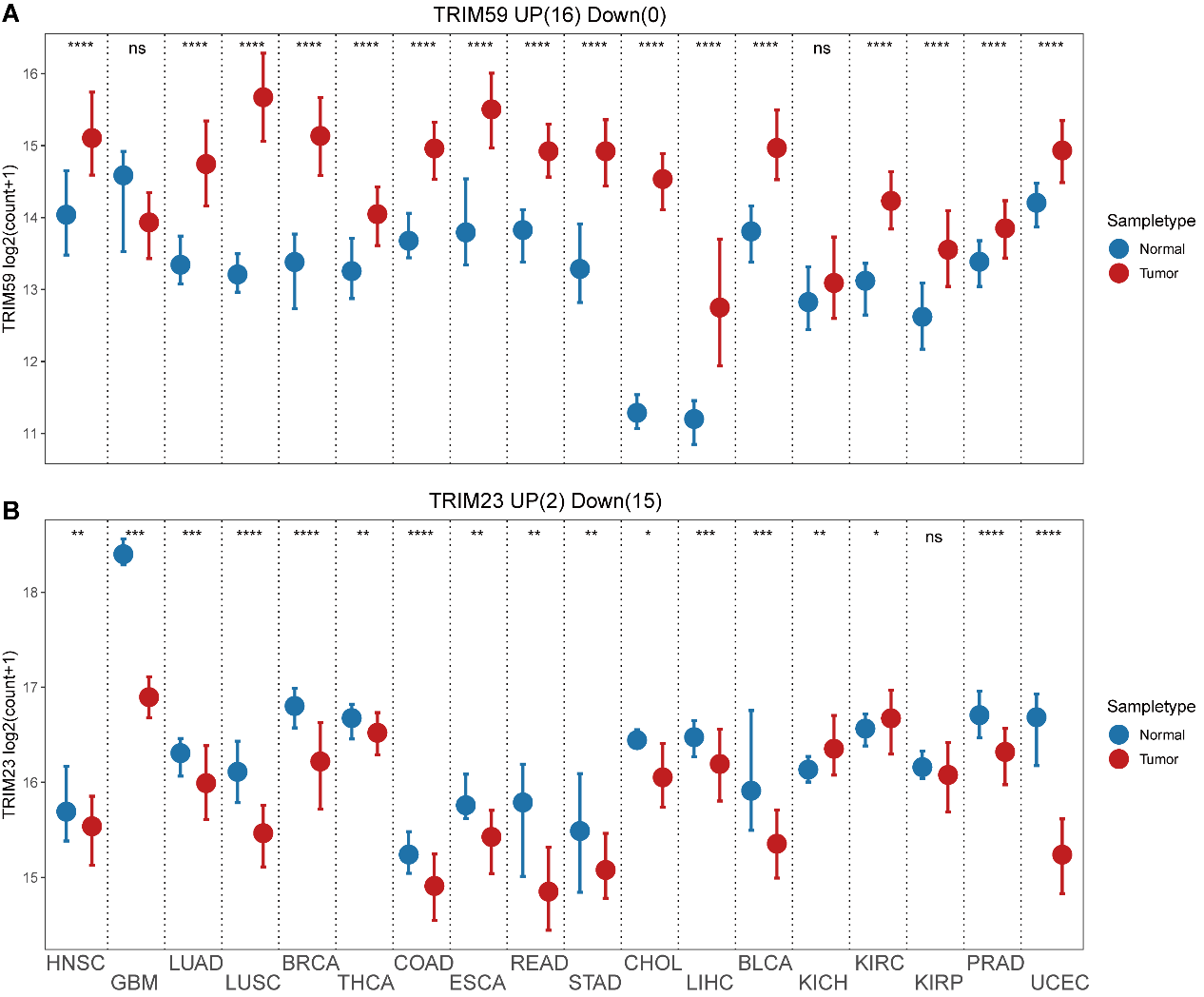


**Fig. S2. Perturbations of the expression of TRIM protein family in cancer.** Box plots showing the expression levels of TRIM59 (A) and TRIM23 (B) between normal and tumor samples across 18 cancer types. The point represents the median value of expression, and sides of the line represent the upper and lower quartiles of that. Wilcoxon rank sum test, **** P<0.0001, *** P<0.001, ** P<0.01, * P<0.05.


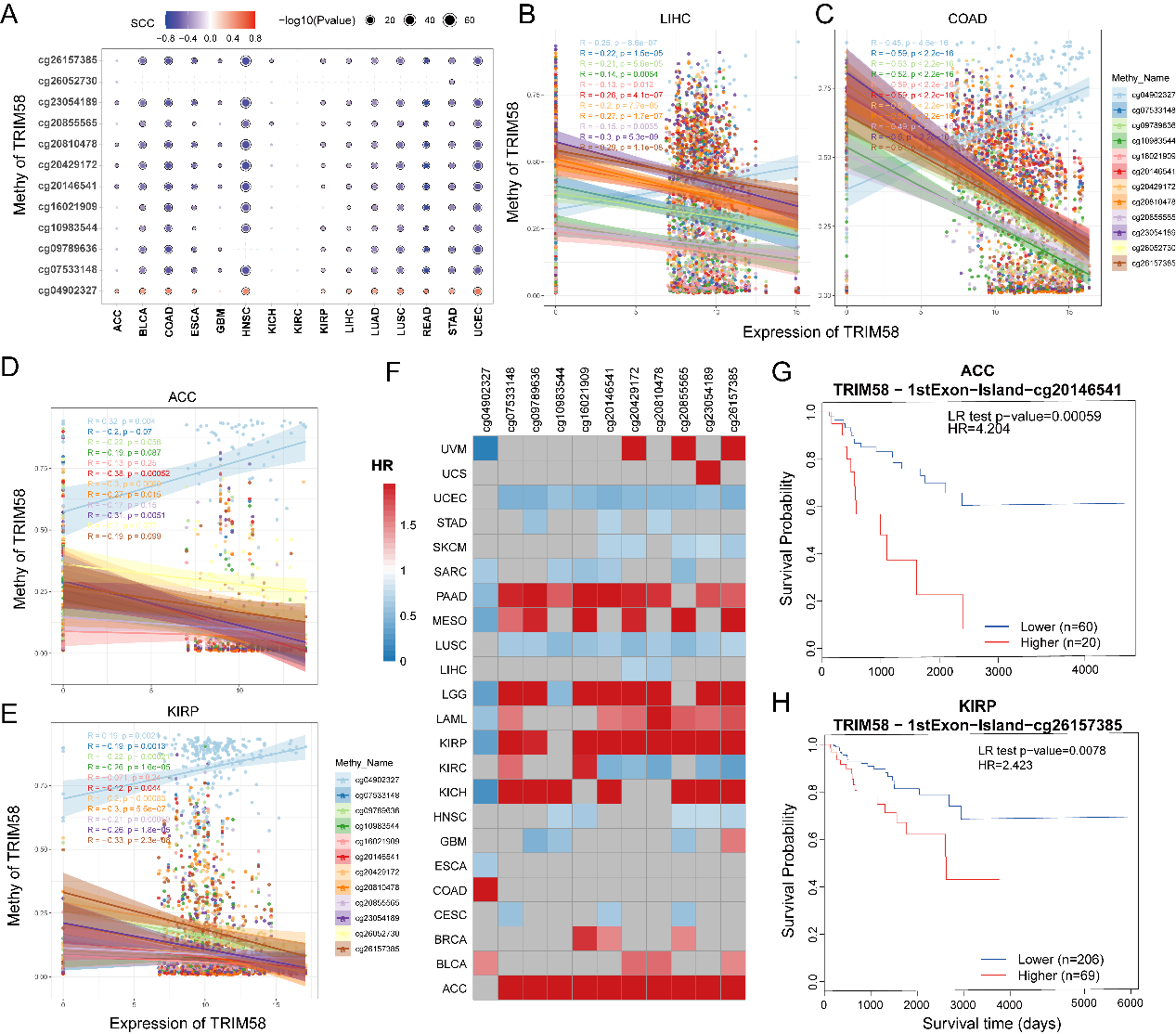


**Fig. S3. Methylation of TRIM58 in various cancers.** (A)The correlation between expression value and methylation beta value of TRIM58 across cancer types. Dot plot showing Spearman correlation coefficient (SCC) and size of the dots corresponding to the -log10(p-values). Red indicates positive, while blue indicates negative. The black border demonstrates significant correlation. (B-E) Scatter plots showing the correlations between expression value and methylation beta value of TRIM58. Dot and lines are colored by methylated subtype. The significant negative correlation has been observed in Liver hepatocellular carcinoma (LIHC, B), Colon adenocarcinoma (COAD, C), Adrenocortical carcinoma (ACC, D) and Kidney renal papillary cell carcinoma (KIRP, E). (F) The distribution of hazard ratios (HR) based on methylated subtype of TRIM58 across different cancer types. Red indicates that high levels of methylation are associated with poor survival (HR > 1), while blue indicates that they are more likely to be protective factors (HR < 1). Grey indicate that the results are not significant (p > 0.05). (G) and (H) Kaplan-Meier survival plot of patients grouped by high vs. low methylation levels using MethSurv platform. Survival curve plot showing that patients with high methylation levels in ACC (G) and KIRP (H) are associated with poor survival (HR >1 and p-value < 0.05).


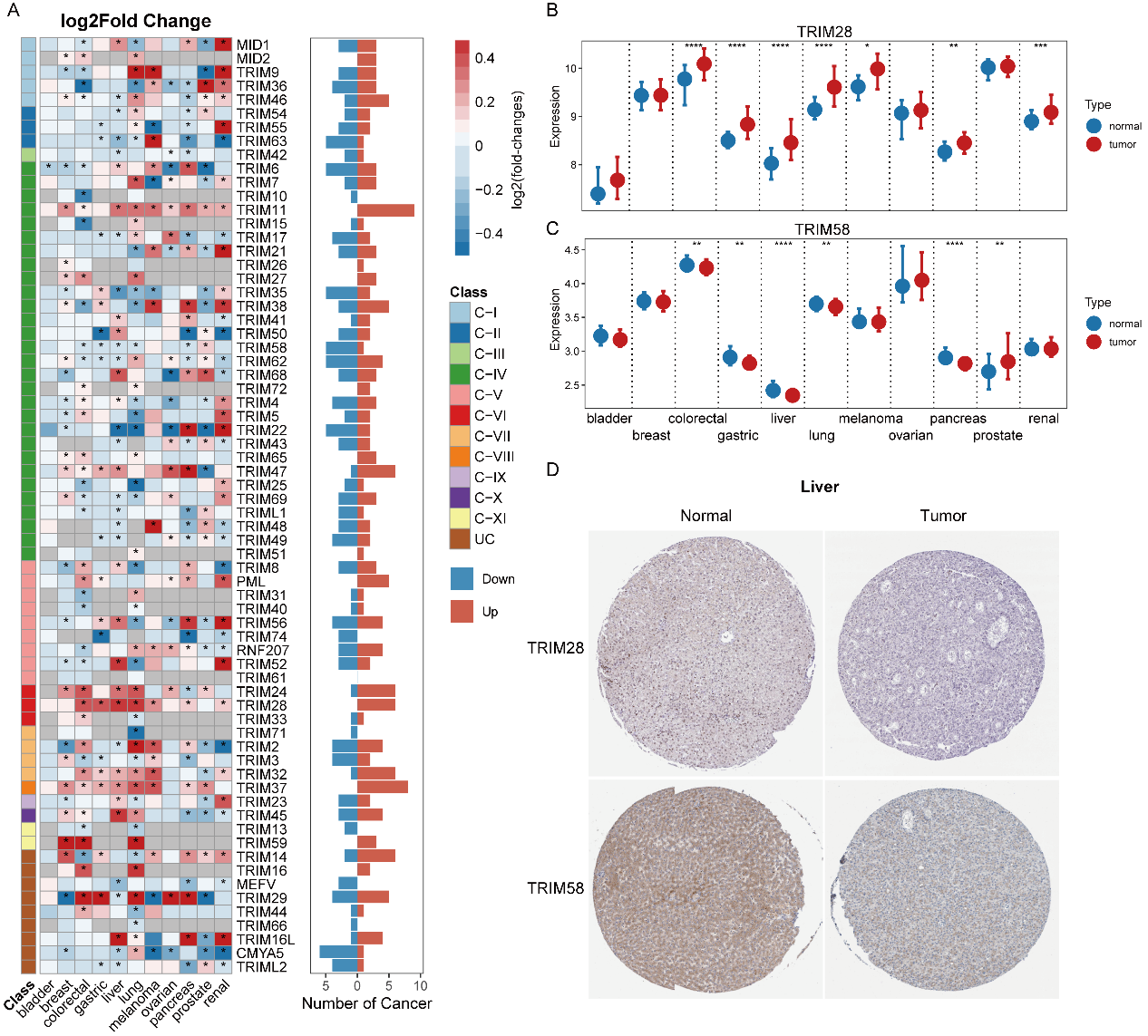


**Figure. S4. Perturbations of the expression of TRIM protein family in additional cohorts.** (A) Landscape of differential expression of TRIM proteins family between normal and tumor samples across cancer types. Heatmap on the left panel showing the log2(fold-changes) of TRIM genes across cancer types, where '*' indicates significant difference. Bar plots on the right panel showing the number of cancers that the corresponding TRIM genes perturbed. Red represents up-regulated expression in cancer, and blue represents decreased expression. (B) and (C) Box plots showing the expression levels of TRIM28 (B) and TRIM58 (C) between normal and tumor samples across 11 cancer types. The point represents the median value of expression, and sides of the line represent the upper and lower quartiles of that. Wilcoxon rank sum test, **** P<0.0001, *** P<0.001, ** P<0.01, * P<0.05. (D) The immunohistochemical results of TRIM28 (top) and TRIM58 (bottom) in Liver tissue from Human Protein Atlas (HPA). The expression of TRIM28 in tumor tissues (HPA064033, Patient id: 3324) is higher than that in normal tissues (HPA064033, Patient id: 3402). On the contrary, TRIM58 expression in tumor tissues (HPA023637, Patient id: 3477) is lower than that in normal tissues (HPA023637, Patient id: 3222).


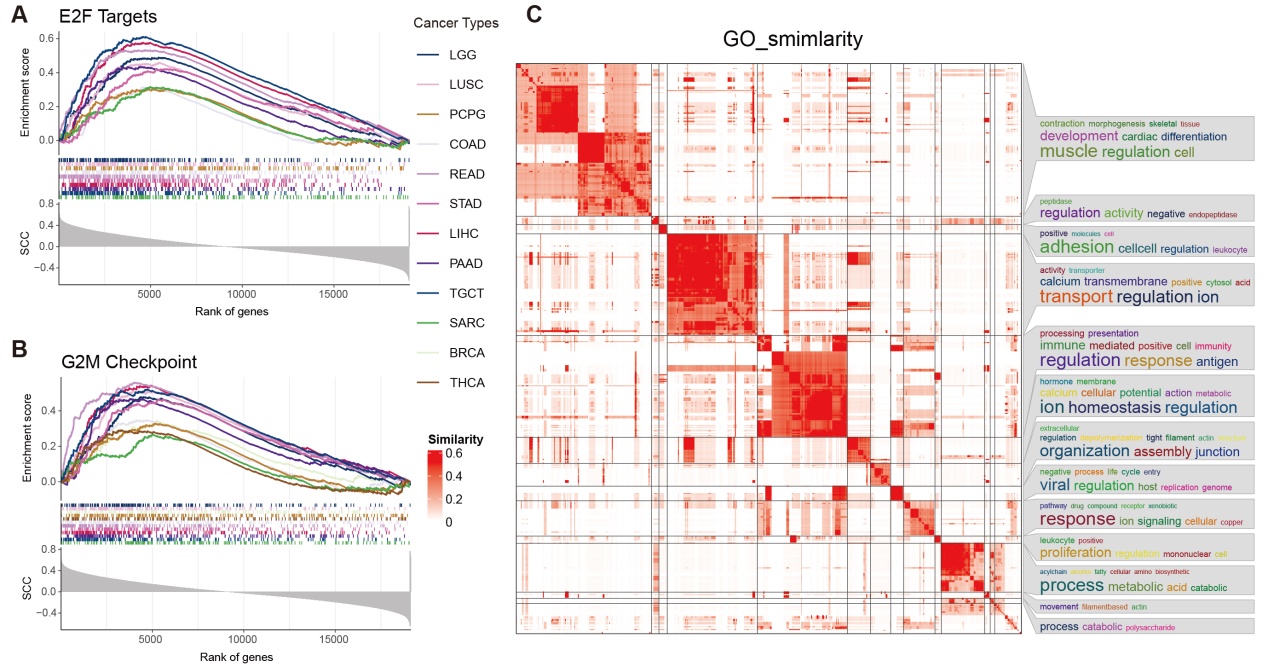


**Fig. S5. Functional pathways of TRIM protein family across cancer types.** (A) and (B) The enrichment score (ES) distribution for the genes positively co-expressed with TRIMs scores in E2F targets pathway (A) and G2M checkpoint pathway (B). Each line is for one cancer and lines are colored by cancer types. (C) Gene Ontology terms enriched by differentially expressed genes. Terms are clustered by similarity of genes.


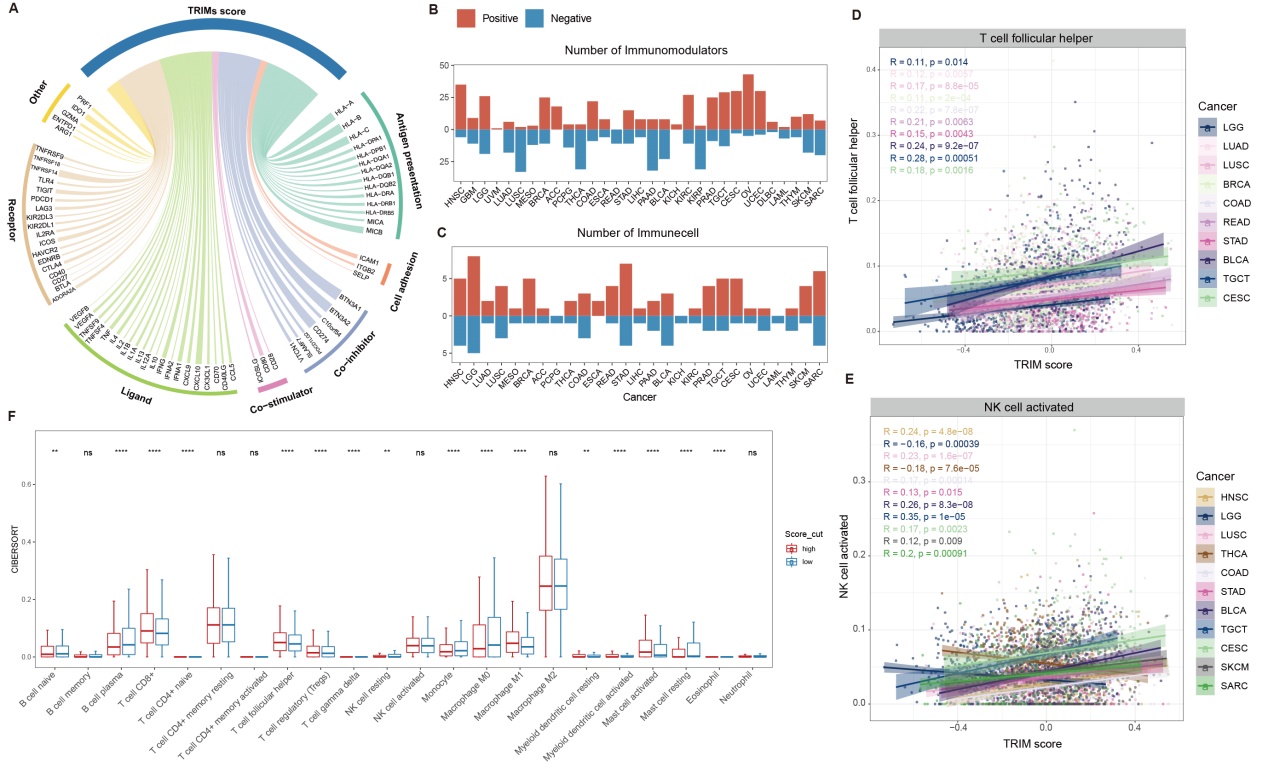


**Fig. S6. Immune regulation of TRIM protein family across cancer types.** (A) Circos plot showing the correlation between TRIMs scores and expression of immunomodulators. (B) Number of immunomodulators correlated with TRIMs scores across cancer types. (C) Number of immune cells correlated with TRIMs scores across cancer types. (D) and (E) Scatter plots showing the correlations between TRIMs scores and T cell follicular helper (D) or NK cell activated (D) infiltrations across cancer types. Dot and lines are colored by cancer types. (F) Boxplots showing the immune cell infiltration levels between TRIMs scores high vs. low groups.


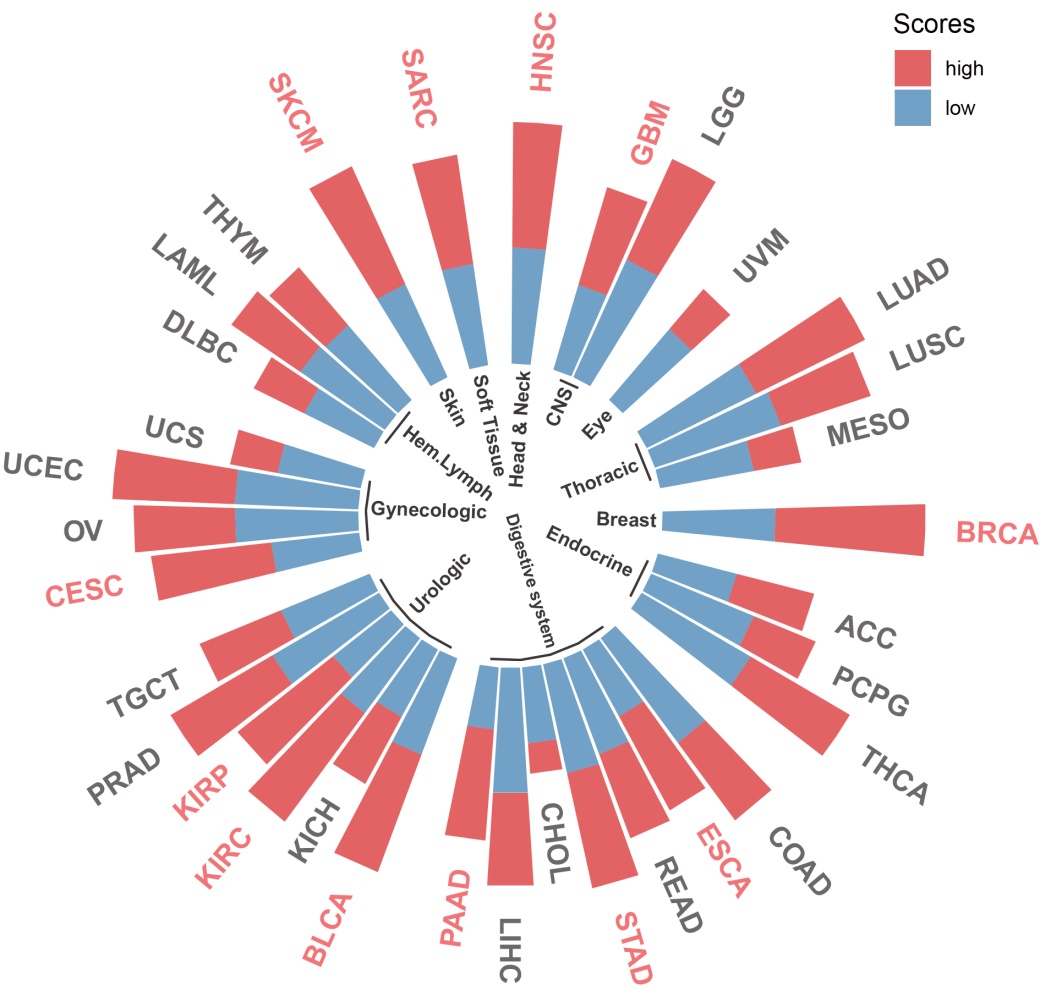


**Fig. S7. Proportion of patients with high or low TRIMs scores across cancer types.**


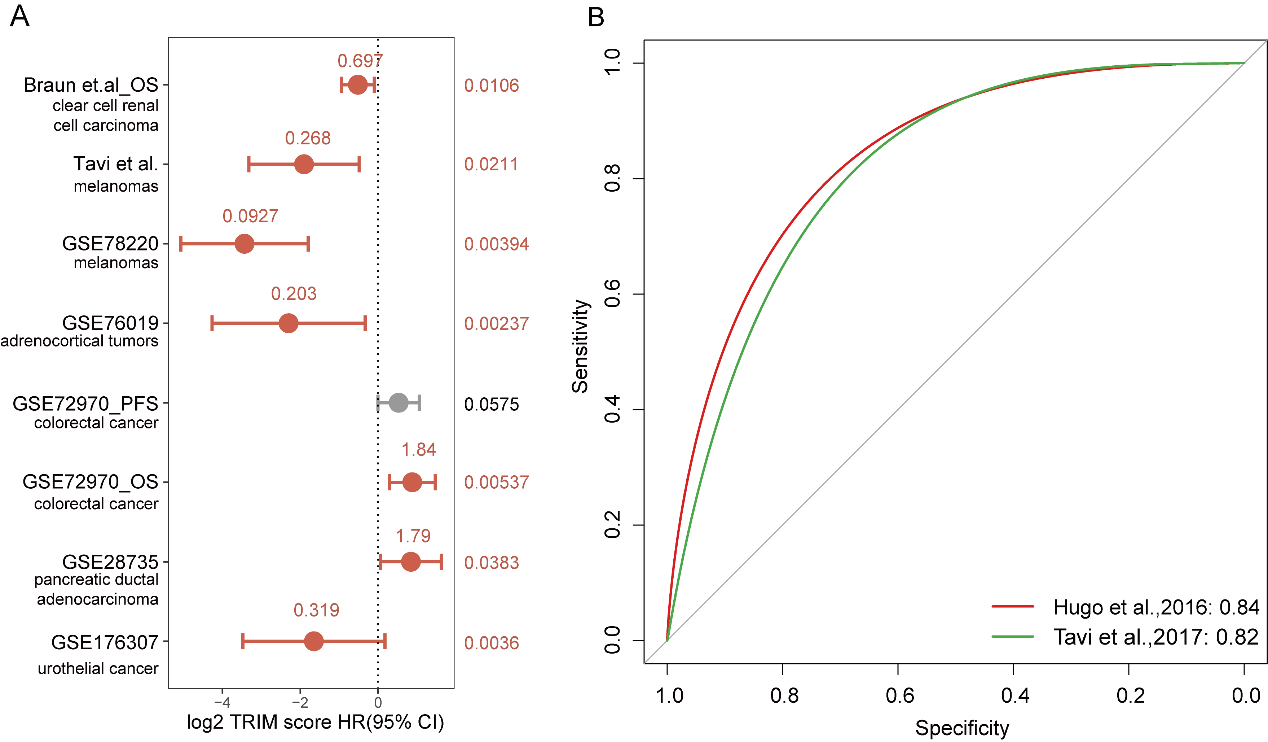


**Fig. S8. Clinical associations of TRIMs scores in additional cohorts.** (A) The distribution of hazard ratios (HR) based on TRIMs scores across different cancer types.The cohort composition (GSE176307, Urothelial Cancer; GSE28735, Pancreatic ductal adenocarcinoma; GSE72970, Colorectal Cancer; GSE76019, Adrenocortical tumors;GSE78220, Melanomas; Tavi et al.(PMID: 27956380), Melanomas; Braun et al. (PMID: 32472114), clear cell renal cell carcinoma) were used in this plot. The point represents the HR value, and sides of the line represent 95%CI. Red dots and lines indicate significant result. (B) Area under the ROC curves (AUCs) for classifiers based on TRIMs scores in anti-PD1 immunotherapy (GSE78220, Hugo et al.) and anti-CTLA-4 immunotherapy (Tavi et al.) cohorts. Legends show the HR value of each cohort. Lines are colored by cohorts in cancer.
